# Multiwavelength metasurfaces through spatial multiplexing

**DOI:** 10.1038/srep32803

**Published:** 2016-09-06

**Authors:** Ehsan Arbabi, Amir Arbabi, Seyedeh Mahsa Kamali, Yu Horie, Andrei Faraon

**Affiliations:** 1 T. J. Watson Laboratory of Applied Physics, California Institute of Technology, 1200 E. California Blvd., Pasadena, CA 91125, USA

## Abstract

Metasurfaces are two-dimensional arrangements of optical scatterers rationally arranged to control optical wavefronts. Despite the significant advances made in wavefront engineering through metasurfaces, most of these devices are designed for and operate at a single wavelength. Here we show that spatial multiplexing schemes can be applied to increase the number of operation wavelengths. We use a high contrast dielectric transmittarray platform with amorphous silicon nano-posts to demonstrate polarization insensitive metasurface lenses with a numerical aperture of 0.46, that focus light at 915 and 1550 nm to the same focal distance. We investigate two different methods, one based on large scale segmentation and one on meta-atom interleaving, and compare their performances. An important feature of this method is its simple generalization to adding more wavelengths or new functionalities to a device. Therefore, it provides a relatively straightforward method for achieving multi-functional and multiwavelength metasurface devices.

Metasurfaces have been of great interest in recent years as they provide optical wavefront control with high spatial resolution, in a thin and flat form factor^1^,^2^,^3^,^4^,^5^. High efficiency, capability to control polarization and phase, and compatibility with the standard nanofabrication techniques have made dielectric transmittarray metasurfaces very attractive for various applications in different parts of the optical spectrum^3^,^6^,^7^,^8^,^9^,^10^,^11^,^12^,^13^,^14^,^15^,^16^,^17^,^18^,^19^,^20^,^21^,^22^. Conventional optical devices such as blazed gratings, lenses, and orbital angular momentum generators^6^,^7^,^8^,^10^,^11^,^13^,^14^,^15^,^16^, simultaneous polarization and phase controllers^12^,^23^, and flexible and tunable devices^18^,^24^,^19^ have been realized with high efficiencies using dielectric transmittarrays.

Similar to other types of diffractive optical devices, metasurfaces which deflect light suffer from large chromatic dispersion^25^,^26^,^27^,^28^. Therefore, these types of metasurfaces are usually designed and operate at a single wavelength. Recently, multiple methods have been proposed for realization of multiwavelength metasurface devices^27^,^28^,^29^,^30^,^31^,^32^. Most of these devices are polarization sensitive and suffer from low efficiencies^27^,^29^,^30^,^31^,^32^.

Spatial multiplexing has been used for enhancing the number of operation wavelengths^33^,^34^ or adding new functionalities to optical devices^35^. Here we show multiwavelength metasurface lenses based on spatial multiplexing with two different approaches: large scale aperture division and meta-atom interleaving. We use dielectric transmittarrays based on amorphous silicon (*α*-Si) to implement the two different methods, and experimentally demonstrate lenses that focus light with wavelengths of 915 nm and 1550 nm to the same distance. The methods introduced here can readily be generalized to more than two wavelengths, or to devices that perform different functions at different wavelengths, or at the same wavelength.

## Concept

**Figure 1:**
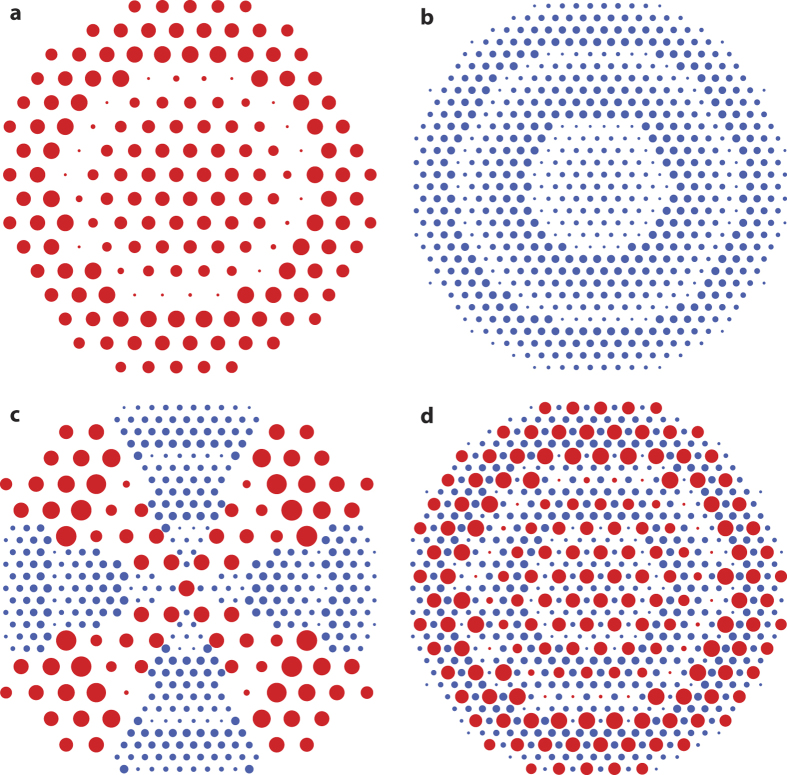
Spatial multiplexing scheme. (**a**) Schematic of a metasurface lens designed to focus light with wavelength *λ*_1_ and (**b**) wavelength *λ*_2_ to a distance *f*. (**c**) Double-wavelength metasurface lens formed by lens aperture division, and (**d**) by interleaving meta-atoms.

Two metasurface lenses designed for two different wavelengths (Fig. 1a,b) can be combined through dividing the metasurface aperture into macroscopic areas, resulting in a multi-sector device as shown in Fig. 1c. A less obvious method is to interleave the meta-atoms of the two lenses (Fig. 1d). If the phase change introduced by the meta-atoms is local (i.e. the coupling between the meta-atoms is small), we can expect light scattered by each group of meta-atoms (one group corresponds to one lens) to interfere constructively in their respective focal spot at the design wavelength.

### Metasurface structure

**Figure 2:**
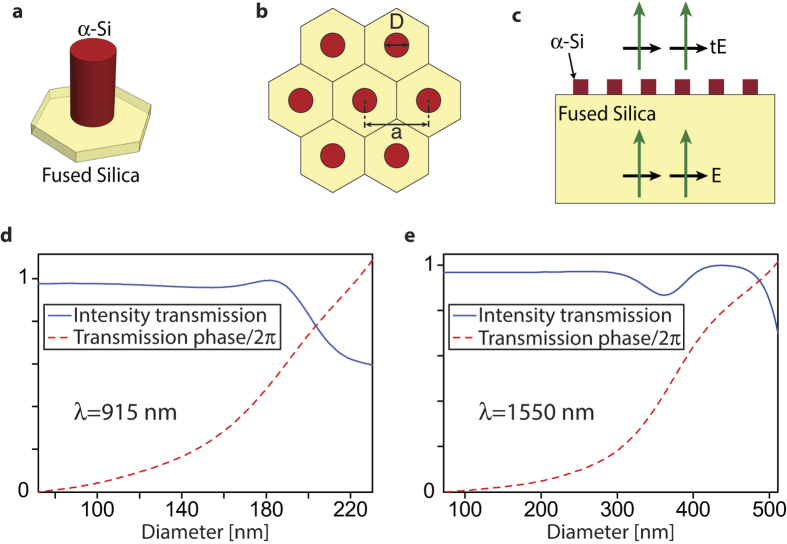
Metasurface structure with simulated amplitude and phase curves. (**a**) Schematic of an amorphous silicon (*α*-Si) cylindrical nano-post on a fused silica substrate. (**b**) Top view of the meta-atoms on a hexagonal lattice showing geometrical parameters. (**c**) Schematic of the simulated structure. (**d**) Intensity transmission, and phase of the transmission coefficient at *λ* = 915 nm and (**e**) at *λ* = 1550 nm. The lattice constant is 360 nm for *λ* = 915 nm and 720 nm for the *λ* = 1550 nm structure. The *α*-Si layer is 718 nm thick in both cases.

**Figure 3:**
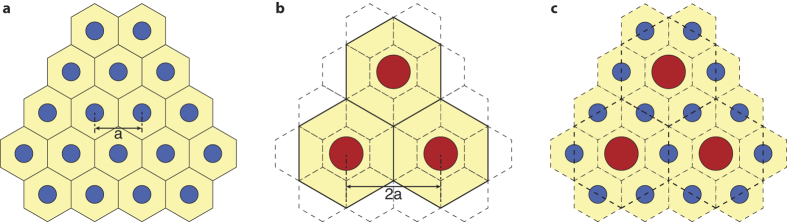
Interleaved lattice schematics. (**a**) Schematic of the short-wavelength metasurface lattice with a lattice constant of *a*. (**b**) Schematic of the long-wavelength lattice with a lattice constant of 2*a*, overlaid on the unit-cell boundaries of the short-wavelength meta-atoms. (**c**) Schematic of the interleaved lattice resulting from replacing one out of four short-wavelength meta-atoms with a long-wavelength one.

We use a high contrast dielectric metasurface platform for implementation of the devices. The platform consists of amorphous silicon (*α*-Si) nano-posts on a fused silica substrate (Fig. 2a) that can form a hexagonal lattice (Fig. 2b). For proper choices of the nano-posts height and lattice constant, full phase coverage can be achieved at a design wavelength by changing the diameters of the nano-posts^11^. The nano-posts behave like multi-mode truncated waveguides with many resonant modes around the wavelength of interest^18^,^36^. Superposition of the scattered fields of these resonant modes can result in full 2*π* phase coverage, while maintaining a high transmission amplitude. Since the structure needs to be fabricated with a single step electron beam lithography, the nano-post heights should be the same at both wavelengths (which we have chosen to be 915 and 1550 nm due to availability of laser sources). In addition, since the two wavelengths are relatively far apart, we choose the 1550 nm lattice constant to be twice that of the 915 nm (Fig. 3a,b). With this choice, the two metasurfaces can be interleaved by simply replacing one out of four 915 nm meta-atoms by a 1550 nm one (Fig. 3c).

Taking these considerations into account, we find that a post height of 718 nm, lattice constants of 360 nm at *λ* = 915 nm, and 720 nm at *λ* = 1550 nm, enable full phase coverage with high transmission at both wavelengths. The simulated intensity transmission 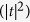 and transmission phase 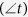 for such uniform lattices are plotted in Fig. 2d,e at 915 and 1550 nm, respectively. For simulations, a uniform array of meta-atoms with a given diameter is illuminated with a plane wave at the wavelength of interest (Fig. 2c), and the transmission amplitude and phase are calculated. We have used the rigorous coupled wave analysis^37^ to perform the simulations.

## Experimental Results

For experimental demonstration, we have designed and fabricated a multi-sector and an interleaved lens that focus light from single mode fibers at 915 and 1550 nm to a focal point 400 *μ*m away from the lens’ surface without spherical aberrations. The lenses are 300 *μ*m in diameter, and the single mode fiber is placed 600 *μ*m away from the backside of the ~500-*μ*m-thick substrate of the lens (resulting in a focal distance of 286 *μ*m, and a numerical aperture of 0.46). The multi-sector lens is formed by dividing two single wavelength lenses designed for 915 and 1550 nm to 8 radial sectors and combining them similar to Fig. 1c and the interleaved lens is formed from combining the two single wavelength lenses in the manner shown in Fig. 3. The single wavelength lenses are designed using the metasurface platforms described above. The smallest nano-post diameter is set to 72 nm in all the designs. For the interleaved lens, a minimum gap of ~50 nm is set between the adjacent nano-posts to facilitate their fabrication. This resulted in the maximum nano-post diameters of 200 and 420 nm for the 915 and 1550 nm lenses, respectively, thus the highest achievable phase delay was ~1.6*π* at each wavelength. The less than 2*π* phase coverage leads to small wavefront errors and lowers the focusing efficiencies of the lenses. In the design process, the best nano-post for each lattice site was chosen by minimizing the complex transmission error defined as 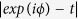, where *ϕ* is the desired phase at the lattice site and *t* is the complex transmission coefficient of the nano-posts. Using this design procedure, and assuming that the lenses require a uniform distribution of nano-posts with various phases from 0 to 2*π*, we find that the incomplete phase coverage achieved here results in a less than 3% reduction in the lens efficiency.

**Figure 4:**
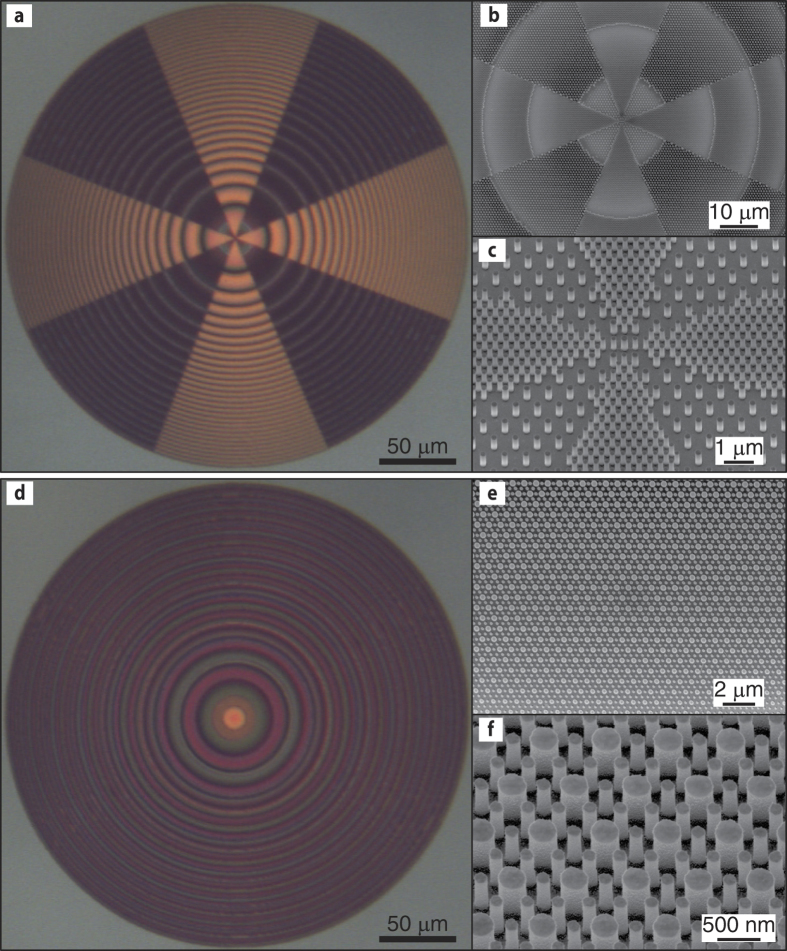
Fabricated device images. (**a**) Optical and (**b**,**c**) scanning electron microscope images of the multi-sector lens. (**d**) Optical and (**e**,**f**) scanning electron microscope images of the interleaved lens.

The devices were fabricated by depositing a 718-nm-thick layer of *α*-Si on a fused silica substrate using the plasma enhanced chemical vapor deposition method. The device pattern was written on an electron beam resist using e-beam lithography, and was transferred to an aluminum oxide layer using a lift-off process. The aluminum oxide layer served as a hard mask for etching the *α*-Si layer in a dry etch process, and was removed in a solution of hydrogen peroxide and ammonium hydroxide. Optical and scanning electron microscope images of both the multi-sector and interleaved devices are shown in Fig. 4.

**Figure 5:**
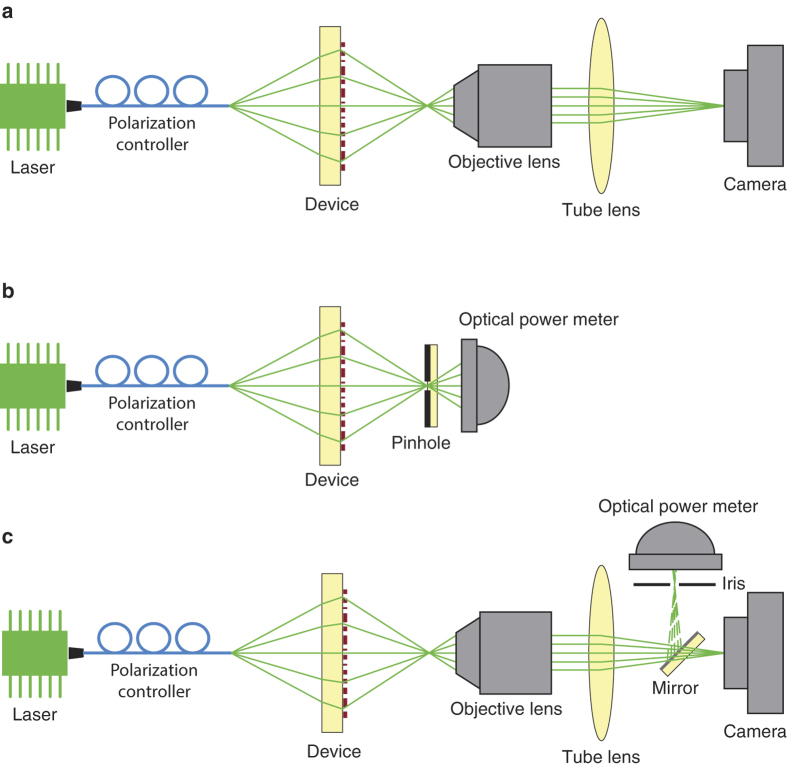
Measurement setup schematics. (**a**) Schematics of the measurement setup used for measuring the optical intensity distribution patterns in different planes at 915 nm. (**b**) Schematics of the setup used for measuring the focusing efficiency of the lens at 915 nm. (**c**) Schematics of the setup used for characterizing the devices at 1550 nm. The flip mirror, iris, and optical power meter were used to measure the focusing efficiencies.

**Figure 6:**
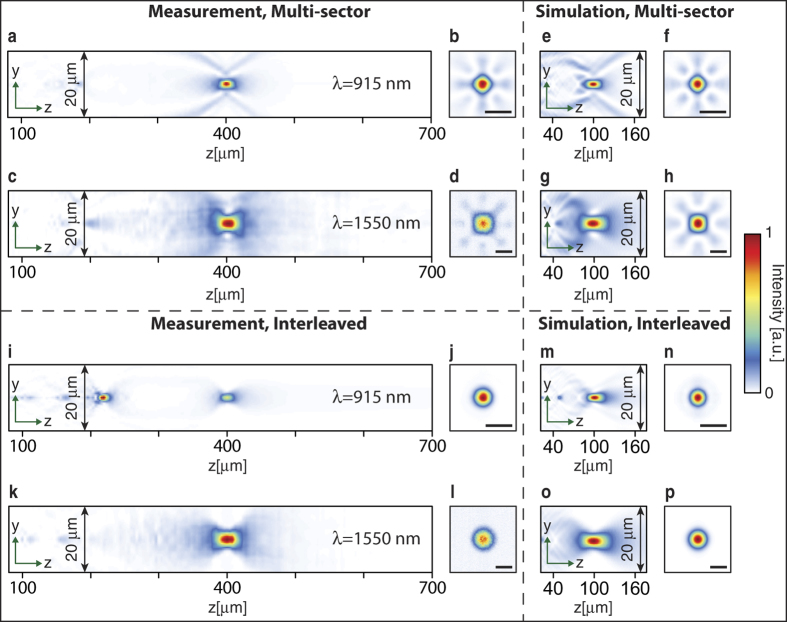
Measured and simulated intensity profiles. (**a**) Intensity measured in the axial plane and (**b**), in the focal plane at 915 nm for the multi-sector lens. (**c**,**d**) Same measurements as (**a**,**b**) at 1550 nm. The high frequency intensity fluctuations observed in the measured focal intensity at 1550 nm is caused by the high non-uniformity in the responsivity of pixels in the phosphorous coated CCD used in 1550 nm measurements. This nonphysical fluctuation is filtered for the axial plane plots to acquire a smoother distribution. (**e**–**h**) FDTD simulated intensities for a similar multi-sector lens with a four times smaller size and focal distance. (**i**–**l**) Similar results to (**a**–**d**), but for the interleaved lens. (**m**–**p**) FDTD simulated intensities for a four times smaller interleaved lens. Scale bars: 4 *μ*m.

The lenses were characterized by measuring the intensity distributions in the focal plane, and in many planes parallel to the focal plane using custom built microscopes with ~100× magnification. Schematics of measurement setups are shown in Fig. 5. Measured intensities in axial and focal planes at both wavelengths are plotted in Fig. 6a–d for the multi-sector and in Fig. 6i–l for the interleaved lens. The polarization of incident light was changed using the polarization controllers shown in Fig. 5, and no polarization dependence was observed. Measured axial plane intensities for the multi-sector lens are plotted in Fig. 6a,c, where a single strong focus is observed at both wavelengths. The intensity distributions in the focal plane are plotted in Fig. 6b,d and show features that are caused by the division of the lens aperture into multiple sectors. The high frequency fluctuations observed in the 1550 nm focal plane measurements are caused by the highly non-uniform responsivity of the phosphorous coated CCD used. To achieve smoother intensity distributions in the axial plane, these high frequency fluctuations are filtered through removing all components with spatial frequencies higher than the free space propagation constant. For comparison, a lens designed with the same method and with the same NA, but with all dimensions and distances four times smaller than the fabricated device was simulated using the finite difference time domain (FDTD) method in MEEP^38^. The smaller size of the simulated device was necessary to make the simulations feasible with the available computational resources. Figure 6e–h show the simulated intensities for this device at both wavelengths in the axial and focal planes. The illumination was linearly polarized in simulations, and the symmetry of the structure ensures the same behavior for other incident polarizations. A very good agreement is observed between simulated and measured focal depths and the focal plane intensities. Focusing efficiency is defined as the ratio of the power focused by the device, to the output power of the fiber, and is measured at 915 and 1550 nm with setups schematically shown in Fig. 5b,c, respectively. The pinhole used at 915 nm has a diameter of 20 *μ*m, and the iris used for 1550 nm is 2 mm in diameter, which translates to a ~20 *μ*m diameter in the object plane. Simulated and measured efficiencies and full width at half maximums (FWHM), in addition to the diffraction limited FWHMs are summarized in Tables 1 and 2, respectively. The diffraction limited FWHMs are found via simulation of a perfect phase mask at each wavelength, with the same optical fiber illuminations that are used to design and measure the lenses.

Measurement results for the interleaved lens are plotted in Fig. 6i–l. Unlike 1550 nm, at 915 nm a second focus is observed at *z* ≈ 220 *μ*m, with the peak intensity approximately 1.8 times, and a power 1.2 times those of the main focus. Similar to the multi-sector lens case, a four times smaller interleaved lens designed using the same platform is simulated for comparison, and the simulated intensities are plotted in Fig. 6m–p. A weak second focus is observed around *z* ≈ 50 *μ*m in the simulations as well. It is worth noting that these devices are multi-order (similar to multi-order gratings), and have multiple focal points (like a Fresnel zone plate lens). These higher order focal points can be seen in all four axial plane measurements in Fig. 6a,c,i,k. The higher order focal points have low intensities in all cases except for Fig. 6i. If the “*blazing*” of the lens is perfect (i.e. the phase profile is equal to its ideal case), all of the power will be directed towards the designed focal distance. However, if some error is introduced to the phase profile, a portion of the power will be directed towards higher order focal points. As this error increases, the power in the main focus will decrease. The 1550 nm nano-posts are optically large and support many resonant modes around 915 nm, resulting in some error in the phase of the total transmitted field at 915 nm. Besides, as we will shortly discuss, the coupling between 1550 and 915 nm posts is not negligible at 915 nm. These errors, result in a significant portion of the power going to the higher order focus at 915 nm for the interleaved lens. Measured and simulated efficiencies and FWHMs are summarized in Tables 1 and 2, respectively.

To determine the origin of the focus observed at *z* ≈ 220 *μ*m, we use paraxial imaging equations by considering the fiber tip as an object and the focal plane intensity as its image. We find that the effective object distance from the lens is 1003 *μ*m using the *f*_1_ = 286 *μ*m focal distance, and the 400 *μ*m image distance. Therefore, the focal distance corresponding to the focus observed at *z* ≈ 220 *μ*m is *f*_3_ ≈ 180 *μ*m. We represent this focal distance by *f*_3_, because there is also a secondary focal distance *f*_2_ = 485 *μ*m arising from the 1550 nm nano-posts, as they also form a lens at 915 nm. The focal distance of the lens formed from 1550 nm nano-posts is given by 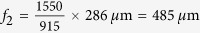 at 915 nm^28^. We note that we have 1/*f*_3_ = 1/*f*_1_ + 1/*f*_2_. This means that the equivalent transmission mask of the lens contains a term proportional to 
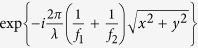
. This term is the result of addition of two phase profiles generated by the 915 and 1550 nm nano-posts, and indicates coupling between these nano-posts (this term cannot exist if 915 and 1550 nm posts operate completely independently). Therefore, at 915 nm the coupling between the interleaved nano-posts cannot be completely neglected. For an optimal design, this coupling can be taken into account if unit cells formed from combining the two nano-post groups are analyzed together^28^.

## Discussion

The efficiency of the interleaved lens at 915 nm is significantly lower than 1550 nm, both in measurements and simulations. Two factors play important roles in this difference between the efficiencies at the two wavelengths. First, the interleaved lens has an effective lattice constant of 720 nm (Fig. 3c), which is close to 
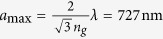
, the lattice constant above which higher diffraction orders will be propagating in the glass substrate at 915 nm (here *n*_*g*_ = 1.452 is the refractive index of glass). Therefore, the non-periodicity of the lens structure results in higher order diffractions propagating inside the substrate. In contrast, this lattice constant is subwavelength enough at 1550 nm such that no higher diffraction orders are present. Second, 915 nm nano-posts are optically small at 1550 nm, whereas 1550 nm nano-posts are optically large and support many resonances around 915 nm. Therefore, while adding the 915 nm nano-posts to the 1550 nm lens results in a relatively small phase error at 1550 nm, introducing the large 1550 nm nano-posts to the 915 nm lens changes the phase profile significantly, and for some nano-post diameters, it also reduces the transmission amplitude. In addition, the measured efficiency for the interleaved lens at 915 nm (10%) is lower than the simulated value of 27%. The transmission phase of the device at 915 nm is more sensitive to errors in the nano-post diameters because of the larger aspect ratio of the nano-posts, and fabrication errors have degraded the phase profile of the lens and its efficiency by directing a significant portion of power to the higher order focus around 220 *μ*m.

Efficiencies of the multi-sector device at the two wavelengths are closer to each other than the interleaved lens. The sum of simulated efficiencies at 915 nm and 1550 nm for these devices is always less than 100%. The interleaved design, in contrary, can have a sum of efficiencies at 915 and 1550 nm higher than 100% as evidenced by the simulation results. This is because the high index nano-posts can have an optical cross-section significantly larger than the geometrical area of the metasurface pixel that they occupy. Besides, the efficiency of the interleaved lens can be increased, and its sensitivity to fabrication errors can be decreased using the meta-molecule concept and a concurrent design of the nano-posts for the two wavelengths^28^. In addition, the division of the aperture to multiple macroscopic sectors changes the shape of the input aperture of the lens and thus the shape of its focal spot. The interleaved design on the other hand, does not cause this issue.

The demonstrated multiwavelength lenses can be used in applications where simultaneous operation at a few discrete wavelengths is required, such as two photon fluorescence microscopy. While conventional refractive achromatic lenses could have a better performance (specifically a higher efficiency) in such applications, they are bulky, more expensive to fabricate, and harder to customize. Besides, multiple metasurfaces can be monolithically integrated in order to correct various aberrations, add functionalities, or be directly integrated with electronics to form compact electro-optical systems^39^. Nevertheless, multiwavelength lenses are demonstrated here only as a proof of concept example for the general method of spatial multiplexing of metasurfaces for implementing multiwavelength multifunctional optical devices. The introduced methods can be directly applied to designing metasurfaces with different functionalities at different wavelengths. For instance, a metasurface can be designed to operate as a lens at one wavelength, and as a grating at the other one. It can also be applied to making metasurfaces that perform multiple functions simultaneously at a single wavelength. It would be very challenging, if at all possible, to fabricate such devices with the conventional refractive optics platform.

## Conclusion

We have shown that by spatially multiplexing metasurface lenses that are designed for operation at two different wavelengths, we can realize lenses that simultaneously operate at both wavelengths. We designed, fabricated, and characterized double-wavelength lenses based on macroscopic aperture division (i.e. the multi-sector lens), and meta-atom interleaving. Although here we used this concept to demonstrate double-wavelength lenses, the idea can be readily generalized to devices with more operation wavelengths, or devices that perform different functions at different wavelengths, or even at the same wavelength. Therefore, spatial multiplexing introduces a simple route towards multiwavelength and multi-functional metasurfaces.

## Additional Information

**How to cite this article**: Arbabi, E. *et al.* Multiwavelength metasurfaces through spatial multiplexing. *Sci. Rep.* **6**, 32803; doi: 10.1038/srep32803 (2016).

## Figures and Tables

**Table 1:** Measured and simulated efficiencies for the multi-sector and interleaved lenses.

Wavelength	915 nm		1550 nm	
	Measured	Simulated	Measured	Simulated
Multi-sector	37 ± 1%	40.6%	30 ± 1%	36.8%
Interleaved	10 ± 0.5%	27%	58 ± 1%	75.8%

**Table 2:** Measured, simulated, and diffraction limited FWHM focal spot sizes for the multi-sector and interleaved lenses.

Wavelength	915 nm			1550 nm		
	Measured	Simulated	Limit	Measured	Simulated	Limit
Multi-sector	1.85 ± 0.05 *μ*m	1.7 *μ*m	1.6 *μ*m	3.3 ± 0.2 *μ*m	3 *μ*m	2.75 *μ*m
Interleaved	1.85 ± 0.05 *μ*m	1.7 *μ*m	1.6 *μ*m	3.3 ± 0.2 *μ*m	3 *μ*m	2.75 *μ*m
